# Critical Problems for Research in Animal Sheltering, a Conceptual Analysis

**DOI:** 10.3389/fvets.2022.804154

**Published:** 2022-04-01

**Authors:** Kevin Horecka, Sue Neal

**Affiliations:** ^1^Research Department, Austin Pets Alive!, Austin, TX, United States; ^2^Arkansas State University, Department of Political Science, Jonesboro, AR, United States

**Keywords:** animal shelters, animal welfare, research problems, animal behavior, shelter adoption, disease transmission, one health

## Abstract

Animal shelter research has seen significant increases in participation over the past several decades from academic organizations, private organizations, public entities, and even corporations that aims to improve shelter programs, processes, operations, and outcomes for the various stakeholders/participants involved in a shelter system (animals, humans, the community, wildlife, and the environment). These efforts are scattered through a huge variety of different research areas that are challenging to define and scope for organizations seeking to start new lines of research inquiry. This work aims to enumerate some of the most critical outstanding problems for research in animal sheltering in a conceptual framework that is intended to help direct research conversations toward the research topics of highest impact (with the highest quality outcomes possible). To this end, we define seven (7) key areas for research: animal behavior, adoptions and special needs populations, medical conditions, disease transmission, community, ecology, and wellness (one health), operations, and public-private-academic-corporate collaboration. Within each of these areas, we review specific problems and highlight examples of successes in each area in the past several decades. We close with a discussion of some of the topics that were not detailed in this manuscript but, nonetheless, deserve some mention. Through this enumeration, we hope to spur conversation around innovative methodologies, technologies, and concepts in both research and practice in animal sheltering.

## Introduction

Animal Sheltering in Western society, in some form, has existed since the mid-1800's (with the creation of both the Royal Society for the Prevention of Cruelty to Animals and the American Society for the Prevention of Cruelty to Animals in 1824 and 1866, respectively) and has been a constantly evolving field to both the benefit (1, 2) and detriment (3, 4), of its stakeholders: animals, pet owners, communities, and the organizations that tie these groups together. In the past several decades, a cultural shift has been occurring in which animal welfare (5, 6) has received more attention, resources, and scrutiny than in the decades before. Success in sheltering is commonly measured by the Live Release Rate (hereafter LRR) that is obtained by dividing the total number of live animal outcomes (such as adoptions and transfers) by the total number of live animal intakes (7). Many cities have been able to increase their LRR and those of surrounding counties above 90 and even 95% (8), yet shelters still struggle with having adequate resources (9) and rural shelters may be more likely to struggle (10). The number of conceptual problems in sheltering is enormous, and as awareness of the needs of shelters continues to rise, more and more groups—academic, corporate, non-profit, and private—are looking for ways to contribute to the wider movement of animal welfare using their unique skills and talents. One difficulty for these potential partners is in understanding what the needs of shelters are and what high-value unsolved problems exist in the field.

Some of these are knowledge problems, others, implementation problems, and even more, systemic, cultural, and societal problems. Almost all require some manner of research to elucidate best practices and truths and differentiate them from traditions and myths. To aid interested parties in contributing to these areas of animal sheltering, we seek to enumerate and explain many of the critical problems for research in animal sheltering so that those organizations and interested parties might find a place to contribute. The key areas for future research were developed through a combination of both empirical and a priori traditions. The empirical approach used included input from animal sheltering professionals, including the responses of over 10 working groups representing more than 300 shelter professionals associated with Human Animal Support Services project to the question of what research needs were to advance animal sheltering. A priori observation and reflection of the researchers and reviews of the existing literature also helped to inform a lengthy list of research needs. These research needs were then thematically grouped in to the 7 key areas. Each of the areas was then evaluated on two factors: the degree of potential impact to animal sheltering and the difficulty in studying the problem.

Table 1 presents the 7 key areas identified. It also presents the impact potential for research in these areas by identifying the top-level impacts that advances in each topic area could have on the field of animal sheltering. While not intended to be all encompassing, this list captures the main topics generated by the authors and the consulted professionals.

**Table 1:** The 7 key problem areas identified in this work and their impact potential in the space of animal sheltering. While not intended to be all encompassing, this list captures the main topics generated by the authors and the consulted professionals.

**Topic area**	**Impact areas**
Animal behavior	Reduce animal surrendered for behavior reasons, increase adoption potential of animals with known behavior issues, increase the likelihood of long-term placement post adoption.
Adoptions and special needs population	Increase lifesaving by finding economically feasible ways to increase the likelihood of placement for special needs animals.
Medical conditions	Increase lifesaving by improving outcomes for animals with medical challenges.
Disease transmission	Reduce suffering and euthanasia associated with transmissible disease. Reduce costs, stress and health hazards for shelters and their workers through novel ways to reduce transmission.
Community, ecology and wellness	Align animal welfare with other social movements aimed at increasing positive outcomes for humans, animals, and the environment.
Operations	Efficient and effective use of available resources. Reliable and valid ways to measure, compare and communicate success. Increase the number of animals that can remain in their homes to reduce shelter intake and improve human/animal welfare.
Public-private-academic-corporate collaboration	Build a body of knowledge practitioners can use. Increase the funding pool for animal welfare initiatives. Draw on the experience and expertise of a broader swath of individuals.

**Figure 1:**
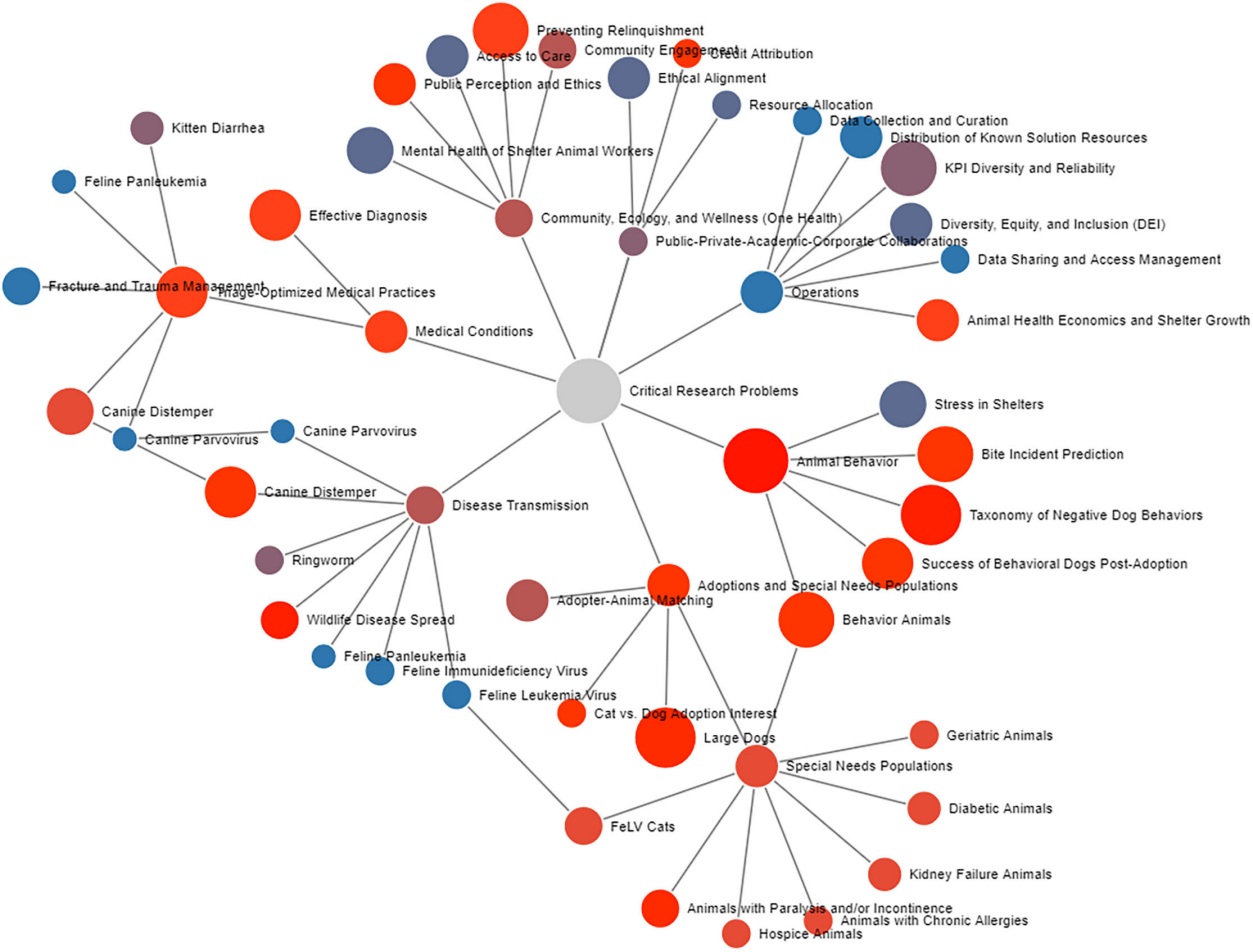
A Force Directed Graph showing the various problems discussed in this article. The size of a node represents the relative impact a solution would have. The color represents the relative difficulty of studying the problem (with red being more difficult). Links relate the topics. An interactive version can be found here: https://codepen.io/kevroy314/pen/jONoXma. Click to isolate nodes and their neighbors. Drag to move around. Scroll to zoom.

Figure 1 provides an additional way of examining these topical areas. This figure provides examples of critical problems for research in animal sheltering and provides a way to compare and evaluate the areas in terms of the relative difficulty of studying the problems as well as the relative magnitude of the potential impact. In addition to evaluating these factors for the key research areas identified herein, this figures also shows a framework through which other researchers could evaluate the relative impacts and difficulty of other possible research topics.

## Key Areas for Research

### Animal Behavior

Animal behavior is one of the most challenging and complex topics in animal sheltering. Leaving aside controversies surrounding the ethics of adopting out animals with known behavior challenges or the ending of the life of an animal, whether for the protection of the public, retribution for an incident, quality of life, or any other justification related to behavioral issues, such as biting or inappropriate elimination, the practical need to better understand and modify animal behavior to improve the lives of animals and their caregivers to improve their chances of adoption and/or their probability of remaining in the home (11) is substantial. Here, we highlight 4 key areas in animal behavior that may have the biggest impact in a shelter setting and that may be underrepresented in the literature.

Efforts to form a typology of dog behaviors that may be problematic in the home, and, specifically, dog behavior that may be averse to a successful adoption and retention in a home (12, 13) have been attempted in the past (14–16). Despite the interest in canine behavior in general rising sharply in the early 1990s and more recently (17), no consensus has been reached upon a singular behavioral classification and identification system that can be used to make decisions around best practices with dogs with histories of behavior problems or potential for behavior problems. Such a classification system should have the following properties [(18) for a more detailed discussion of the difficulties surrounding some of these issues]:

- Objective measurability and reproducibility.
- Characterization of common temporal progressions.
- Understood correlations between related behaviors.
- Clinical relevance to predictability and intervention.

Somewhat recent attempts (18, 19) at assessing the efficacy of behavioral evaluations have not been as promising as might be hoped given the 50+ year history of the field, and the impact of such a system, especially in establishing new interventions that can help these animals be successfully placed in homes, could be enormous given the extreme difficulty in achieving successful outcomes for dogs with behavior issues.

One key factor in negative animal behaviors, especially as pertaining to the adoptability of animals, is the stress they experience while in a shelter setting (20–22). Studies of animal stress date back to 1926 (23) with animals have often serving as a model for human stress (24, 25). Practical tools are needed to assess the impact of shelter environmental improvements. Using Biomarkers to assess stress (26) across species (27–29) have shown significant success in recent years. Unfortunately, the practical measurement of such biomarkers in shelter settings remains unlikely due to resource and practical constraints. Non-invasive measures of stress are possible in many species [including thermographic (30, 31), salivary (32), visual (33), and multimodal (34) systems], though their efficacy as an intervention target is unclear. A more thorough understanding of best practices around the reduction of stress for animals in shelters will allow for significant improvement in the quality of life of long-stay animals as well as the adoptability of animals that may show fewer behavioral issues once removed from a stressful environment, with some evidence showing changes in cortisol levels, a common biomarker for stress, with even a single night removed from the shelter environment in adult dogs (35).

A key element in the success of an animal with behavioral issues, post-adoption is not simply the cessation of negative behaviors, but also the match with an adopter who can maintain the environment necessary for permanent improvement in behavior as well as following up with those adopters to ensure continued success is achieved. Preparing adopters and proper matching is key given the frequency of post adoption behavioral issues among shelter animals (36). This problem comes down to two key sets of questions:

Given evidence suggesting choice of pet is often tied to factors like appearance more than behavioral considerations (37), how should shelters best match behavioral issues with potential adopters who can handle the maintenance surrounding those issues to reduce the chance of return (36, 38, 39) and adverse incidents such as bites or escape from a yard?

What risks (i.e., environment impact on biting) (40) exist in the home that might exacerbate issues surrounding behavior?

Finally, when it comes to animal behavior, especially canine behavior, one of the most critical incidents that can occur is a bite incident since these can result in serious injury to persons and potentially result in liability claims against the shelter (41). The previously mentioned issues all likely contribute to the probability of a bite incident occurring, but predictions of such events, even in aggregate across a city (42), are challenging at best. A successful bite prediction system would also pose ethical issues as individuals, shelters, and cities may choose to use such a system to decide which animal's lives should be preemptively ended, to avoid the potential risk and liability. It is critical, therefore, that the predictability of bite incidents increase at the same rate as our ability to reasonably intervene to prevent the incidents.

### Adoptions and Special Needs Populations

The core problem with adoptions at shelters is always “how do we get as many animals out to good homes as quickly as possible?.” Of course, as with so many seemingly simple problems, the posing of the question in such a general manner means no obvious solutions present themselves. Properly reframing the question often begins to imply solutions. Preventing the surrender of animals to the shelter system is certainly a key component to assuring positive outcomes for animals and people alike. New programs, such as the Human Animal Support Services project, are focusing heavily on programs aimed at keeping animals out of the shelter altogether and in their original homes whenever possible. Further, this paradigm shift has the potential to profoundly impact positive outcomes for community cats, who may not be best served through adoption. Although adoption is not the only possible positive outcome for all animals that enter a shelter system, for many animals (and the humans who manage the systems of sheltering), it remains an important practical and ethical outcome. Here, we review 3 key areas in adoptions that remain complex and difficult despite extensive efforts in the sheltering community. For a more complete list of these challenge areas, see the American Pets Alive! Documentation on the topic (“American Pets Alive! Resources;” https://americanpetsalive.org/resources) (43).

First and most critically, large dogs, often considered to be those weighing over approximately 35 pounds or 16 kilograms, consistently have more difficulty in being adopted (44–46). This can be due to factors such as the general public perception around larger breeds (47, 48), city ordinances banning ownership of certain breeds (49), housing restrictions implemented at the facility level (50), or concerns around safety, behavior, and compatibility with other home residents (51). These issues are exacerbated by the difficulty in accurately identifying breed information in shelter animal populations (52). As a result of these complications around getting large breeds out of shelters, shelters often end up with a stagnant population of these animals that has less turnover than other, easier to adopt categories (puppies of any breed, for example). This can create a perception that the only populations present are these large breed animals. These factors result in many of these animals having long stays and, as mentioned in prior sections, increased stress and overall wellness difficulties that further worsen their adoptable potential. Moreover, animals in the shelter are less likely to behave the way they might otherwise in a home (53), further decreasing their chances for a positive outcome. A strategy around breaking this cycle and helping large dogs would alleviate significant amounts of trapped resources as site maintenance and housing can create substantial costs and reduce flexibility in serving other populations. The importance of providing an equal opportunity for these large breed dogs to stay in their home is one consideration beyond adoption in strategy design. For example, policies that disallow the use of size of a dog as criteria for access to housing (as discussed above) would help keep these animals out of the shelter system in the first place. Adequate access to resources to behavior training could be another community level intervention that could allow more of these animals to stay in their homes.

#### Other Special Populations

Beyond these major issues, there are numerous conditions of decreasing commonality that require increasingly complex adaptations of program and policy to accommodate. This article cannot enumerate all such conditions, but the following list, sorted roughly by difficulty, captures some of the most critical special needs populations that require specially trained homes to inhabit, making them more difficult to adopt out:

- Geriatric Animals.
- Animals with Chronic Allergies.
- Hospice Animals.
- Feline Leukemia Virus (FeLV) Cats.
- Kidney Failure Animals.
- Diabetic Animals.
- Behavior Animals.
- Animals with Paralysis and/or Incontinence.

Much of the care, maintenance, and treatment of these populations is well understood, but the problem of placing them in amenable homes is still a significant one. More research around interventions that can increase the likelihood of placement as well as the factors that impact the likelihood of special population placement may provide actionable insights [see (54) as an example in geriatric animals].

Finally, and significantly, a more thorough understanding of how to match adopters to animals (37, 55), how to evaluate homes for safety and longevity of adoption outcomes (13, 56), how to optimize placement of animals in homes (57), and what preferences exist when it comes to adoption practices around marketing, visitation, and engagement is desperately needed. This understanding will likely depend significantly on local cultural distinctions in populations (58, 59) and is, therefore, difficult to examine systematically. More best practices around adoption matching and marketing would greatly simplify one of the most critical functions in animal shelters.

#### Unique Challenges of Cats

Another, potentially less obvious problem in sheltering is the difference in positive outcomes for cats vs. dogs. Best Friends, a national non-profit that provides the most comprehensive summary of annual shelter statistics reports that cats are still dying in shelters at a ratio of 2:1 when compared with dogs (60) despite approximately one-fourth of US households providing a home for cats (61). Many shelters consistently report difficulties in adopting out adult cats once they no longer have the appearance of a kitten (62). Further, shelter or municipal policies around the extermination of community cats (63) may also be a significant contributor to the numbers of cats not having successful outcomes in shelters.

Approaches to improving live outcomes for cats require shelters to explore ideas outside of the traditional intake to adoption framework. Some strategies that are specifically applicable to cats have been evaluated and shown to be effective such as trap-neuter-return and shelter-neuter-return, which could reduce the number of un-adoptable cats entering the shelter system (64, 65), but more research into the social drivers and potential interventions for this issue are warranted. A development of the recognition of the ecology of community cats is an additional issue that is elaborated on in Section Operations.

### Medical Conditions

In addition to its capacity as an adoption agency for unowned animals, animal shelters often perform a variety of medical services. These services depend on the location, resources, and risk tolerance each organization has, and it is often difficult for organizations to decide what to treat and what to not treat (whether euthanasia is then called for or not). One critical element of this that remains a challenge for all shelters is the effective, actionable diagnosis of disease [see, (66)]. Many diseases have reliable tests (such as canine parvovirus) while others have a much more complicated history in the development of a reliable test [such as canine distemper, though many strongly claim RNA tests should be considered reliable; (67–69)]. Cost is also a critical factor in shelter tests as even a relatively inexpensive (50 dollars) test in an outbreak scenario can be entirely impractical in a population of just a few dozen animals. Further research into low-cost testing is certainly needed for a wide variety of diseases.

Once the disease is identified, shelters often lack the resources for what would be considered “standard” care in a private practice. Some shelters opt to not offer reduced care and, instead, euthanize, while others choose to offer whatever care they can within their own ethical limitations of suffering and quality of life considerations. The need for significantly more research into evidence-based medical guidelines, and especially those that are specifically optimized for triage situations with limited resources and around medical conditions seen in shelters, is widely apparent. Some conditions, such as kitten diarrhea, may be somewhat understood in a general medical sense, but the treatments and time course do not scale appropriately for the model of a medium to large shelter.

Although many diseases could use additional scrutiny for the purposes outlined above, the following are of particular interest due to the costs, in either lives or resources, associated with typical treatment or management (T; indicates specific transmissible disease relevant to Section Disease Transmission):

- (T) Canine parvovirus (70, 71).
- (T) Feline panleukemia (72).
- (T) Canine distemper (73).
- (T) Feline leukemia virus (FeLV) (74).
- (T) Feline immunodeficiency virus (75–78).
- Kitten diarrhea (79).
- Fracture and trauma management.

### Disease Transmission

More so than the treatment of disease, the prevention of disease spread in the shelter environment is one of the most challenging, concretely measurable in the form of infection rates, yet ambiguous (difficult to diagnose in source) tasks a shelter may face. Shelters are examples of anthropogenic biological instability due to the housing of transient, displaced mixed-species of animals that may not have prior veterinary care or have been scavenging during times of homelessness (80). The disease transmission in shelters is further complicated by situation of overcrowding, poor levels of hygiene, and housing of multiple species which can add significant sources of stress for the animals and create a perfect environment for pathogen emergence and transmission (80). This transmission can quickly lead to a crisis in the shelter (81). Shelters that treat infectious disease like the canine parvovirus establish isolation areas in which only that disease is treated, but little is known about the ease with which these diseases spread under different quarantine practices.

Although there are many interesting diseases that are typically seen in shelters, some (such as those listed in Section Medical Conditions) are considered more impactful/deadly than others and, therefore, would make excellent targets for more detailed studies of disease spread.

While it is not officially recommended as a best practice (82), when shelters experience disease outbreaks, some may opt to depopulate, i.e., end the lives of their entire population, (83) rather than have it persist through many generations of animals flowing through the system. Better understanding of how to stem these outbreaks rapidly, efficiently, safely, in a resource-efficient manner, and given the constraints of a shelter environment (space, staffing, facility design, and the need to maintain normal operations) will allow shelters to avoid mass culling and take an approach that increases lifesaving with more confidence.

### Community, Ecology, and Wellness (One Health)

Beyond the scope of the basic operations of a shelter in managing the conditions of individual animals and placing them in appropriate homes, shelters also serve a critical role in the community as providers of services that can enhance public perception and wellbeing (84). This collaboration requires an engaged community that recognizes the importance of animal welfare in the health and wellness of the larger, shared space. Best practices around establishing this type of engagement are not well identified in the existing body of knowledge. This is further confounded by variation in the distribution of resources and community attitudes in different geographic areas.

As animal shelters continue to evolve in response to societal shifts in attitudes toward animals, the focus of operations are changing from centering on adoptions to centering on the prevention of surrender of animals to the shelter in the first place [see (11) for a review]. This has already been discussed as it relates to community cats and behavior/health but there are many other human-centered reasons that animals are surrendered to shelters such as guardian health problems, housing insecurity, domestic violence, and many others (85). Our understanding of how human welfare intersects with animal welfare has the potential to have a dramatic impact on the way shelters operate in their communities. Some communities have hotlines, spay and neuter programs, and other medical/behavioral services that can potentially contribute to this issue, but the efficacy of such systems and the gaps they leave are not well understood. More significant study of the needs of local populations as they relate to shelter success is needed.

Local populations also differ in their perception and support of shelter policies, ethics, and the local system of laws that are intertwined with these efforts. No unified system of ethics is established in animal sheltering, and communities often do not understand the nuances of practices in shelters (especially regarding resource allocations and euthanasia practices). This makes galvanizing community support difficult, even in communities that have achieved remarkably high live release rates. Public perception, messaging, and ethical alignment will undoubtedly continue to be an ever-evolving socio-cultural landscape that is sorely in need of attention.

The mental health of volunteers, staff, and veterinarians (86) in animal shelters also requires much more attention than it often receives. Individuals that participate in euthanasia are reported to have higher work stress and lower job satisfaction than their counterparts (87). Suicide rates are significantly higher in the field of animal welfare than other high-stress fields (88, 89), and more understanding and support is needing to help those working in these areas receive the help they need to continue to serve the community in a sustainable, healthy manner.

#### Access to Care

Access to veterinary care is emerging as a critical issue in animal welfare. Access to care is an aspect of the One Health approach to considering animal welfare due to the zoonotic potential of various diseases that can find reservoir in companion animals (90). In addition to being a risk to public health, lack of access to veterinary care can result in surrender of animals to shelters, stress to the caregiver/family (91) as well as stress to veterinarians who must counsel caregivers who cannot afford the recommended care (92). Shelters feel the impact of this as downstream recipients of animals when owners surrender due to an inability to access needed care. This can both drive surrender to shelters and result in a greater financial burden for shelters to meet medical needs that may be complicated by a historic lack of access to preventative or early intervention care. Further, shelters themselves compete in the market to employ veterinary professionals and support staff that may be further complicated by a shortage of veterinarians (93, 94).

Access to care can be seen as a problem with multiple causes from cost to lack of transportation to the unequal distribution of veterinary resources across the landscape. Cost was identified as the most common barrier to accessing veterinary care in the Access to Veterinary Care Coalition report on this issue (91). In the past decade, costs for veterinary care have been outpacing increases in human health care (95). The average American spends 47% more on equivalent veterinary care today than a decade ago (96). The functional impact of this increasing cost is that fewer people are seeking care for their pets (97) resulting in what is considered the greatest current threat to companion animal welfare in the US (91). More research that identifies efficient, effective, and sustainable solutions to the cost of veterinary care will be key for animal shelters.

Key research questions in access to care can come down to three key areas:

Advances in areas like incremental care or spectrum of care, which are not equivalent but present different perspectives on the issue of cost-benefit analyses in treatment protocols, could reduce costs and prevent shelter surrenders but could also help shelters mitigate the increasing expense of medical treatment for animals in their care.

A deeper understanding of the number of animals surrendered for medical reasons, the types of these conditions and potential treatment routes pre-surrender would also add valuable knowledge to the animal sheltering and animal welfare communities.

Development of community-based solutions that focus on disease prevention when the cost is likely lower than when a disease process is more advanced. This includes the prevention of infectious disease transmission in the community and the development of effective education around other preventable conditions by pet guardians.

#### Ecology/Environment

The study of the ecology surrounding community cats has received significant attention over the past several years (63, 64, 98–102), and debates are likely to continue in this area to determine the most effective ways to ensure the health and safety of community cats and the organisms with which they interact. Additionally, ecological perspectives on the interaction between stray and roaming animals in general and the community are also of interest, but often only actively studied due to concerns over infectious disease spread such as the Rabies virus. Finally, the interaction of wildlife systems with domesticated animals may be of some interest both due to the spread of infectious disease and the more complex interactions these two animal groups may have with one another.

As animal welfare incorporates a One-health approach, further research that identifies strategies to reduce the environmental impact of shelter operations cannot be ignored. Effective ways of cleaning outdoor kennels without contributing to contaminate run-off, ecological disposal of animal waste and the evaluation of how large-scale animal transport can contribute to environmental degradation are just a few examples of the interaction of sheltering and the environment that are open for additional exploration.

### Operations

In addition to the study of animal-centric, adoption-centric, and community-centric aspects of sheltering, the study of the operations that contribute to the ability of shelters to continually adapt, and advance is of critical importance if we are to have systems robust to disaster and capable of implementing our values and ethics on a global scale. Although blueprints do exist that can guide communities in setting up new shelters and enhancing existing shelters, significant problems remain in the space beyond the distribution of known solution resources. Here, we discuss 4 key operations problem areas with varying levels of complexity.

#### Data Problems

Shelters need to collect data to know how they are serving their animals, adopters, volunteers, staff, and community, and how to improve operations in all areas of the shelter. While the industry recognizes the need for quality data, significant barriers have been identified such as a lack of training and resources [(103), additionally, see the Associate for Veterinary Informatics (AVI) for additional information on this topic; https://avinformatics.org/]. Solutions such as ShelterLuv, Chameleon, and PetPoint for database management go a long way to improving situations for shelters, but the ability to flexibly collect and curate all manner of useful data (including electronic medical records, location-based event history, and other meta-data about entities that comprise shelters) remains an open problem. It is also essential that the prioritization and understanding of the critical importance of data is shared by line staff as well as senior management. When line staff fail to understand the importance of complete data collection this action can be de-prioritized in fast paced shelter environment.

Beyond this, shelters need methods of protecting themselves in the sharing of data with the public, academic institutions, and each other. The public, which support shelters through taxes or donations, show widespread support, for example, for programs that reduce levels of shelter euthanasia shelters (104). The best practices around of performing data sharing and managing data access for shelters have yet to be established (though some progress has been made in recent months at the Municipal Shelter level). Over time, there have been attempts to create a single authoritative collection of sheltering data but to date, none have achieved high success. The current initiative that has achieved the most progress is Shelters Animals Count (SAC). SAC is a national database that relies on the voluntary participation by shelters and animal rescues to upload monthly sheltering summary statistics. Unfortunately, there is still relatively poor participation. For example, in 2020 there was participation by only 422 municipal shelters, 359 private shelters and 516 rescues (105). This can be contrasted with a 2014 estimate by the Humane Society of the United States of 3,500 municipal and non-profit shelters and over 10,000 rescue organizations (106). Despite the move toward increasing transparency in government, only a small handful of states and municipalities require reporting to their state and local governments, with even fewer providing enough clarity as to what should be reported for such reporting to be of use to the wider sheltering community. The result of this paucity and irregularity of data provides a significant challenge to researchers and policymakers in understanding what is happening across the nation regarding sheltering, though the contributions of states in which reporting is mandated effectively have provided a valuable starting point for these efforts.

#### KPI Problems

Once data is collected, linking that data down to trackable KPIs (Key Performance Indicators) that are useful to shelters in improving outcomes for animals is a challenge in and of itself. The standardization of KPIs and their strict definitions has suffered from some of the disagreement and difficulties surrounding data collection. The most marked attempt to create unified KPIs occurred in 2004 resulting in the Asilomar Accords. The Live Release Rate, and methods of fairly but consistently calculating it materialized as a critical outcome of the Accords (107). This measure has never been without controversy and is limited, in part, by the wide variance in the various ways in which animal shelters operate in their community and what their priority services are (108). As the operation of shelters have changed, with more innovative programs designed to prevent animals from ever entering the shelter system appearing, advancements in medical and behavioral interventions, and the geographically biased nature of animal population distributions (109), the use of a single KPI will likely remain a source of both conflict and difficulty for many shelters. A more diverse set of KPIs will allow for shelters to perform more nuanced comparisons of their successes and failures that will enable better sharing of solutions and resources. What this list of KPIs should entail remains an open problem [see (110)].

#### Growth Problems

Finally, as some shelters begin to stabilize the animal welfare situation in their cities, adapting to the varying degrees and paces of growth in various organizations to ensure resources are being properly utilized to the benefit of animals and the community is a challenge, to say the least. The field of Health Economics in humans has a rich history (111), and a similar field in Animal Health Economics (112, 113) will likely need to be expanded beyond its traditional focus on production animals so that organizations are not put in a position to blindly guess at the proper allocations or resources toward different intervention programs (such as a canine parvovirus treatment program, FeLV treatment program, behavior program, or kitten foster program).

One particularly challenging program area for shelters to understand in the context of growth, integration, and resource allocation is the management of foster programs. Foster programs have been fantastically successful as a method of expanding the effective capacity of shelters, increasing live outcomes (114), enhancing community engagement, increasing quality of life of animals in care (35), and providing special assistance for more difficult to adopt populations. However, a thorough understanding of how to best engage, utilize, and grow foster programs is lacking.

#### Diversity Equity and Inclusion

Researchers have evidenced that the oppression of non-human animals, disabled humans, and people of color are deeply interconnected (115). If animal shelters are to continue to function as key members of diverse communities it is essential that they pay increasing attention to issues of diversity, equity, and inclusion in their operations both internal and external. While the community of research in this space has assembled a basic understanding of some inequities that currently exist, many others have yet to be explored in a thorough way. For example, we know that African Americans are underrepresented in leadership positions (116). The homogeneity of animal shelters is not confined to the workforce alone. Two large survey-based studies found similar results in evaluating the demographics of animal welfare volunteers concluding that most volunteers were White females in the middle to upper middle class (117, 118). Questions of why this lack of diversity persists and what successful strategies could be used to improve conditions would be of benefit as representation of communities within organizations that serve them allow those organizations to supply the appropriate services to maximize the community benefit and foster a highly participatory, engaged, fair, enthusiastic, and ethical social system.

Beyond direct engagement with shelters as volunteers or employees, there are fecund areas for research in the provisioning of shelter operations. As increases in public-private partnerships place more animal shelters in the business of providing animal control operations, the enforcement of ordinances becomes a key issue in balancing public demand for action and the ethics and priorities of animal welfare. A recently published commentary on the subject argues that there is inherent bias in the design and enforcement of public policy around animal welfare and urges a shift from enforcement to resource provision (119). Evaluating policies and enforcement and implementation of these policies and whether biases are leading to unequal burden are not well understood though it is difficult to not draw comparisons to the arena of policing and the long, complicated relationship between marginalized communities and law enforcement personnel. Additional challenges persist in understanding potential inequities between the surrender of animals and the adoption of animals and whether these differences enforce equity imbalances or are based on existing biases and structural inequities (120).

### Public-Private-Academic-Corporate Collaborations

A less visible and virtually unstudied problem in animal sheltering is the ability for organizational entities of different types and with different incentives to collaborate to the benefit of animals, their owners, the community, and each other. The social network analysis of Reese and Ye (121) is a prime example of the complex collaborative relationships that can emerge between organizations to advance lifesaving in a community. Many questions in this space exist around the best ways for these organizations to interact (i.e., what roles are best served by what organizations, what incentives are best to ensure ethical treatment of all parties, and what restrictions should be put on various types of interactions). Legal restrictions around the use of shelter animals in research may be a barrier that exists to research collaborations between shelters and academic institutions. Dialogue, consensus, and potential legislative change may be needed between animal shelters, the veterinary community, and academia to address the negative consequences of legislation originally intended to protect animals from harm.

Public-private partnerships in other areas of medicine have become increasingly common and valuable (122), and corporate sponsorship of shelters has become increasingly common. Public-private shelter partnerships are also on the rise with some proposing this structure as the new standard in the field (123). Academic collaboration with animal shelters, where academic institutions take advantage of the wealth of available subjects and data in shelters, is still a relatively new concept. Though many potential pitfalls exist in these collaborations (including issues with credit attribution, resource allocation, and ethical alignment), the potential to accelerate the state of the art in animal sheltering via these collaborations is huge thanks to the varied strengths of each organizational type.

## Discussion

The seven key areas for research in animal sheltering outlined above are not the only areas that might be of interest to shelter practitioners and their partners. Some additional areas of interest were not mentioned specifically in this manuscript due to the well-researched nature of the topics, the lack of clear definition in the space, and/or their relative distance from the typical practices of an animal shelter. These areas, nonetheless, merit some mention due to their importance to the area of animal welfare research at large and potential intersection with some shelter practices (depending on specific shelter policy, philosophy, and operations).

A variety of interventions have been proposed that might address some of the problems mentioned in this manuscript. On the behavior side, playgroup services have been proposed that may aid in social development and lead to more positive behavioral outcomes for dogs (124). Moreover, foster programs that take advantage of these and other medical or behavioral services to accelerate positive outcomes for animals deserve significant attention (35, 125). Foster programs can serve as an additional reservoir for animal populations, increase community engagement in the shelter system, and encourage positive outcomes for animals in the foster system through positive environmental enrichment in homes. In situations where foster homes are not available, additional environmental enrichment to achieve similar aims may be found through clever building and facility design at the shelter site (126, 127). Finally, a variety of programmatic and procedural interventions around lost and found animals, self-service rehoming, and intake-to-placement optimization, and field services optimization that aim to prevent animals from entering the physical shelter facility can serve as systems optimizations that improve outcomes for all parties; though, more research is needed in these areas to examine their efficacy. Each of these intervention areas, and other innovations in sheltering, deserve significantly more attention than can be afforded in this outline, and future work should attempt to address them more directly.

In addition to a variety of community and ecology problems and interventions, ethical problems in the industry of animal sheltering are not specifically addressed in this work as these are not research topics *per se*, but more in the realm of philosophy. Future work should examine ethical questions surrounding the topics outlined in this manuscript and other sociological research questions related to the ethics of animal shelter practices.

In this work, we present a conceptual organization of topics for research in Animal Sheltering. These topics vary significantly in difficulty and impact but represent a large swath of needed scientific contributions in the literature. Many of these areas are being actively worked upon by various research institutions (i.e., significant work in animal diseases has occurred), but some have received little attention yet (i.e., operations research). Moreover, some of these areas are being examined, but due to resource and/or methodological constraints, progress is slow. By enumerating these problems, the community of researchers attempting to improve the function of shelters for animals, staff, volunteers, and the community can more carefully and wholistically consider the breadth of applicability of their ideas and investigations and hopefully, more productively contribute to the literature.

## Author Contributions

The above document was drafted and originated by KH with review, added sections on community cats, DEI, and Access to Care and most edits provided by SN. Figures were generated by KH. Both authors contributed to the article and approved the submitted version.

## Funding

Funding for publication was provided by the American Pets Alive! Organization, while funding for individual authors and the creation of the work herein was provided by their respective institutions or the authors themselves.

## Conflict of Interest

The authors declare that the research was conducted in the absence of any commercial or financial relationships that could be construed as a potential conflict of interest.

## Publisher's Note

All claims expressed in this article are solely those of the authors and do not necessarily represent those of their affiliated organizations, or those of the publisher, the editors and the reviewers. Any product that may be evaluated in this article, or claim that may be made by its manufacturer, is not guaranteed or endorsed by the publisher.
